# Resveratrol alleviates bleomycin-induced pulmonary fibrosis via suppressing HIF-1α and NF-κB expression

**DOI:** 10.18632/aging.202420

**Published:** 2021-01-20

**Authors:** Zaiyan Wang, Xiaoning Li, Hao Chen, Li Han, Xiaobin Ji, Qiubo Wang, Li Wei, Yafang Miu, Jing Wang, Jianfeng Mao, Zeming Zhang

**Affiliations:** 1 Department of Respiratory Medicine, Shanghai University of Medicine and Health Sciences Affiliated Zhoupu Hospital, Shanghai 201318, China

**Keywords:** pulmonary fibrosis, treatment, resveratrol

## Abstract

The absence of a gold standard for treating pulmonary fibrosis makes its management challenging. We established a rat model to study the effect of resveratrol (Res) on bleomycin-induced pulmonary fibrosis. Rats were randomly divided into control, model, low-Res, middle-Res, high-Res, and dexamethasone groups and treated with various concentrations of these drugs. Rats showed typical features of pulmonary fibrosis; i.e., alveolitis, fibrous hyperplasia, and fibrosis on days 7, 14, and 28, respectively. Expression of HIF-1α and NF-κB was higher in the middle-Res, high-Res, and dexamethasone groups than in the control group, but was less than that in the model and low Res groups. We conclude that different levels of HIF-1α and NF-κB expression at different stages of pulmonary fibrosis in rats is positively correlated with the disease severity. Furthermore, resveratrol alleviates bleomycin-induced pulmonary fibrosis by suppressing HIF-1α and NF-κB expression, indicating its potential as a promising therapeutic drug candidate.

## INTRODUCTION

Pulmonary fibrosis is an interstitial lung disease characterized by early alveolar injury, inflammation, and late lung fibrosis. The fact that it is caused by a combination of different factors makes its prognosis worse and results in recurring lung injury. Moreover, continuous secretion of various cytokines results in chronic inflammatory reactions. The repair process, initially beneficial, eventually results in disorganized deposition of overproduced collagen and scarring of lung tissue and thus fibrosis. Despite the increasing incidence of pulmonary fibrosis each year, its clinical treatments are limited. Therefore, current research is focused on finding effective therapeutic targets.

Hypoxia-inducible factor-1 (HIF-1), an oxygen-dependent transcription factor discovered by Semenza et al. [1] in 1992, binds to a specific sequence in the hypoxia response element (HRE) to initiate the transcription of downstream genes. HIF-1 exists as a heterodimer, consisting of a 120 kD alpha subunit (HIF-1α) and 91/93/94 kD (three molecular weights) beta subunit (HIF-1β). HIF-1α, the regulatory and active subunit of HIF-1, maintains the intracellular oxygen homeostasis under hypoxic conditions. Moreover, it functions as an upstream transcription factor for vascular growth signaling pathways [2, 3]. Elevated expression of HIF-1α in alveolar epithelial cells in bleomycin (BLM)-induced animal models and human pulmonary fibrosis indicates its involvement in the pathogenesis of this disease [4, 5].

Nuclear factor-κB (NF-κB) is a redox-sensitive transcription factor that can cause DNA damage or lipid peroxidation and regulates the expression of several genes involved in inflammation, apoptosis, and proliferation. Moreover, it is known to be involved in causing acute lung injury [6]. Although NF-κB is highly expressed during the onset of pulmonary fibrosis [7], its levels change in different stages of the disease. Resveratrol is a non-flavonoid polyphenol compound. Studies have shown that resveratrol has a therapeutic effect on tumors, pulmonary fibrosis, and other proliferative disorders [8–11]. However, the effects of intervention with resveratrol on pulmonary fibrosis have not been fully explored.

We studied the expression of HIF-1α and NF-κB during different stages of pulmonary fibrosis and the intervention effect of resveratrol on pulmonary fibrosis in rats.

## RESULTS

### Resveratrol reverses bleomycin-induced pulmonary fibrosis *in vivo*

**Figure 1:**
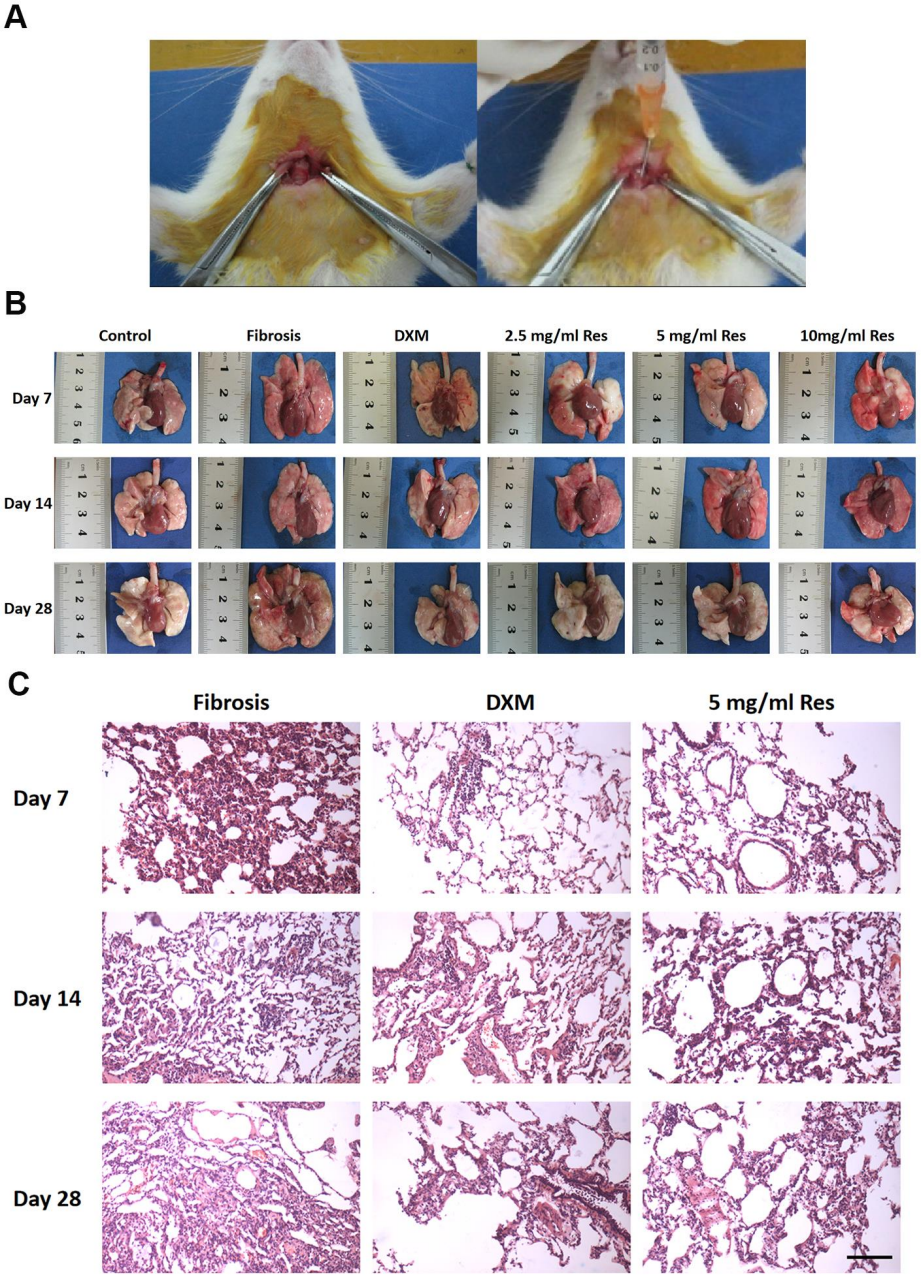
**Resveratrol reverses bleomycin-induced pulmonary fibrosis *in vivo*.** (**A**) Administration of resveratrol to establish bleomycin-induced pulmonary fibrosis model in rat. (**B**) Gross views of the lungs on days 7, 14, and 28 following different treatments. Data are expressed as mean ± standard deviation (SD) of three independent experiments. *p < 0.05, **p < 0.01, ***p < 0.001, ****p < 0.0001. (**C**) The results of hematoxylin and eosin (H&E) staining of samples obtained from the lungs on days 7, 14, and 28 following different treatments (scale bar = 200 μm).

We first successfully established a BLM-induced pulmonary fibrosis model in rats by administering BLM (5mg/kg) intrathecally (Figure 1A). After the rats were sacrificed, the lungs were studied for physical appearance. The lungs appeared the same at each time point in the control group—ruddy with a smooth surface, soft and uniform texture, and no bleeding spots on the surface. In the model group, the lungs appeared dark red on day 7, with increased volume and scattered bleeding spots on the surface. The lungs were gray and white on day 14, with a few nodules of varying sizes and old bleeding spots. The lungs turned pale on day 28 with reduced volume and increased hardness. Nodules and rope-shaped grooves, with partial consolidation, were present on the surface. The observations in the low-Res group were the same as those in the model group. In the middle-Res group, the lungs were dark red on day 7, with slightly increased volume and scattered bleeding spots on the surface. The lungs were slightly red on day 14 with old bleeding spots; there was no change in the volume of the lungs on day 28. The surface was not smooth and had small scattered nodules. There was no hardening and consolidation. The observations in the high-Res and dexamethasone (DXM, positive control) groups were the same as those in the middle-Res group (Figure 1B). Histological analysis using hematoxylin and eosin (H&E) staining was performed to study the pathological changes in the lungs. Alveolar inflammation was observed on day 7 in the model and low-Res groups. Numerous inflammatory cells were observed exuding the alveolar cavity, with a small amount of collagen deposition in the lung interstitium. Pulmonary fibrosis, with a small degree of cell infiltration, was noticed on days 14 and 28. Compared with the model and low-Res groups, pathological changes in the lung tissues of rats in high-Res, middle-Res, and DXM groups were less prominent (Figure 1C).

### Resveratrol exerts beneficial effects on pulmonary fibrosis

**Figure 2:**
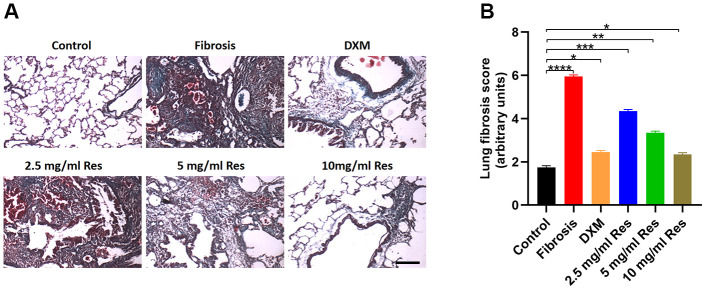
**Histological analysis reveals the beneficial role of resveratrol on pulmonary fibrosis.** (**A**) Masson’s trichrome staining of lung samples in different groups on day 28 post operation (scale bar = 200 μm). (**B**) Lung fibrosis scores in the four groups (*n* = 6 in each group). *p < 0.05, **p < 0.01, ***p < 0.001, ****p < 0.0001.

Masson’s trichrome staining revealed thin collagen fibers in the bronchial wall in the control group, with a small amount of collagen fiber deposition between the alveolar walls. In the model and low-Res groups, collagen fibers were largely distributed around the bronchi and small blood vessels, with no visible collagen fibers in the alveolar space on day 7. Collagen fibers around the bronchi and small blood vessels increased on day 28. Several interstitial collagen fibers were observed to be distributed in sheets and bundles, with some alveolar cavities even filled with collagen fibers. Compared with the model and low-Res groups, less prominent pathological changes in lung tissues were observed in middle-Res, high-Res, and DXM groups (Figure 2A). Statistical analysis of these findings indicated that resveratrol improved the lung fibrosis score (Figure 2B).

### Resveratrol alleviates the negative effects of bleomycin on Beas-2B cells

**Figure 3:**
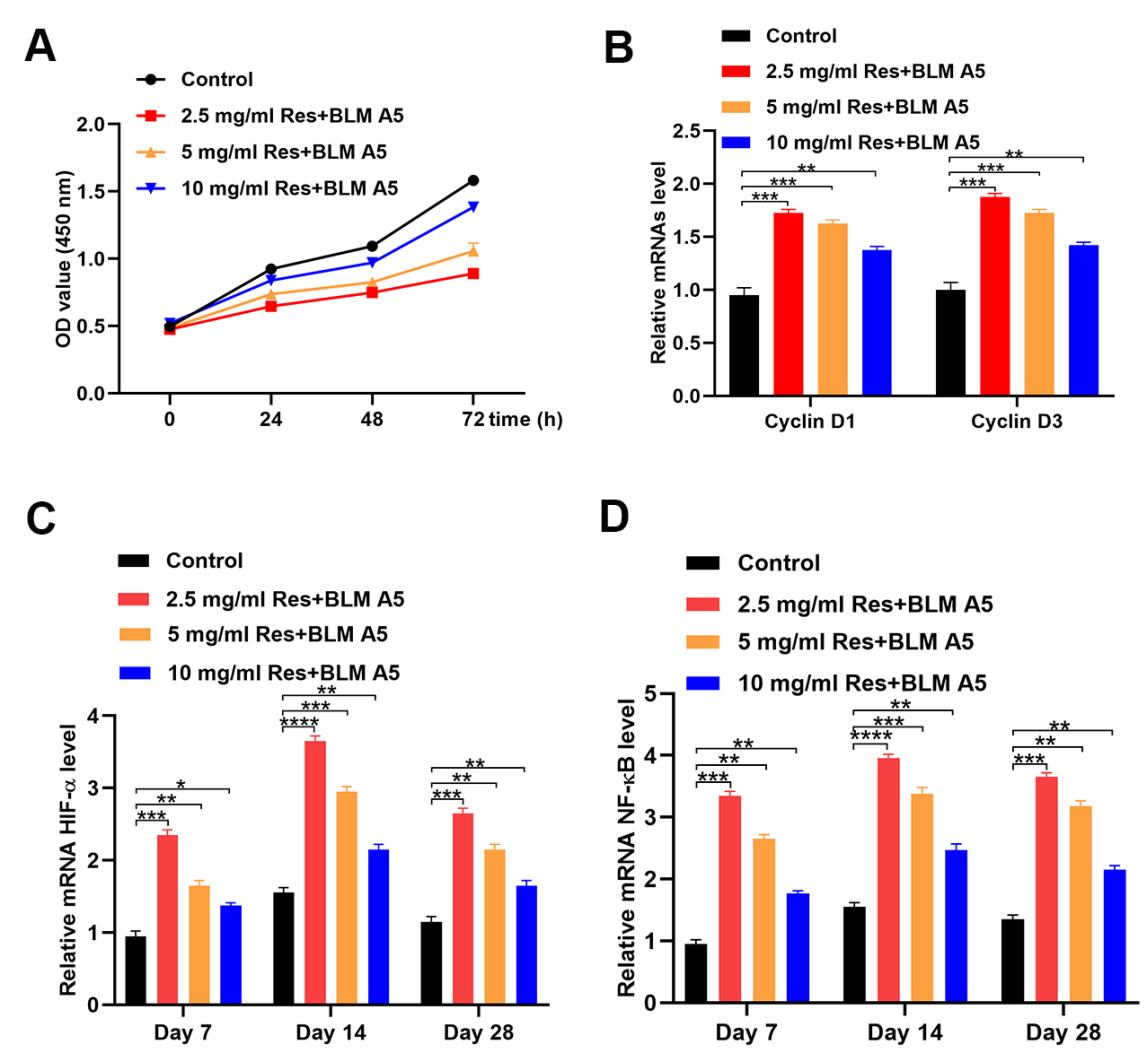
**Resveratrol alleviates the effect of bleomycin on BEAS-2B cells.** (**A**) The cell viability of BEAS-2B cells after different treatments (*n* = 6 in each group). (**B**) The expression of Cyclin D1 and Cyclin D3 in different groups (*n* = 6 in each group). (**C**) The level of HIF-1α in different groups at each postoperative time point (*n* = 6 in each group). (**D**) The expression of NF-κB in different groups at each postoperative time point (*n* = 6 in each group). *p < 0.05, **p < 0.01, ***p < 0.001, ***p < 0.0001.

As shown by the CCK-8 proliferation assay, the proliferation of Beas-2B cells decreased with an increase in the resveratrol dose (Figure 3A). We next determined the expression of Cyclin D, a proliferation-related gene, following different treatments, and observed that resveratrol reduced Beas-2B cell proliferation (Figure 3B). Moreover, the qRT-PCR results showed a negative correlation between the expression of HIF-1α and NF-κB and resveratrol dose (Figure 3C, 3D).

### HIF-1α is overexpressed in pulmonary fibrosis in rats

**Figure 4:**
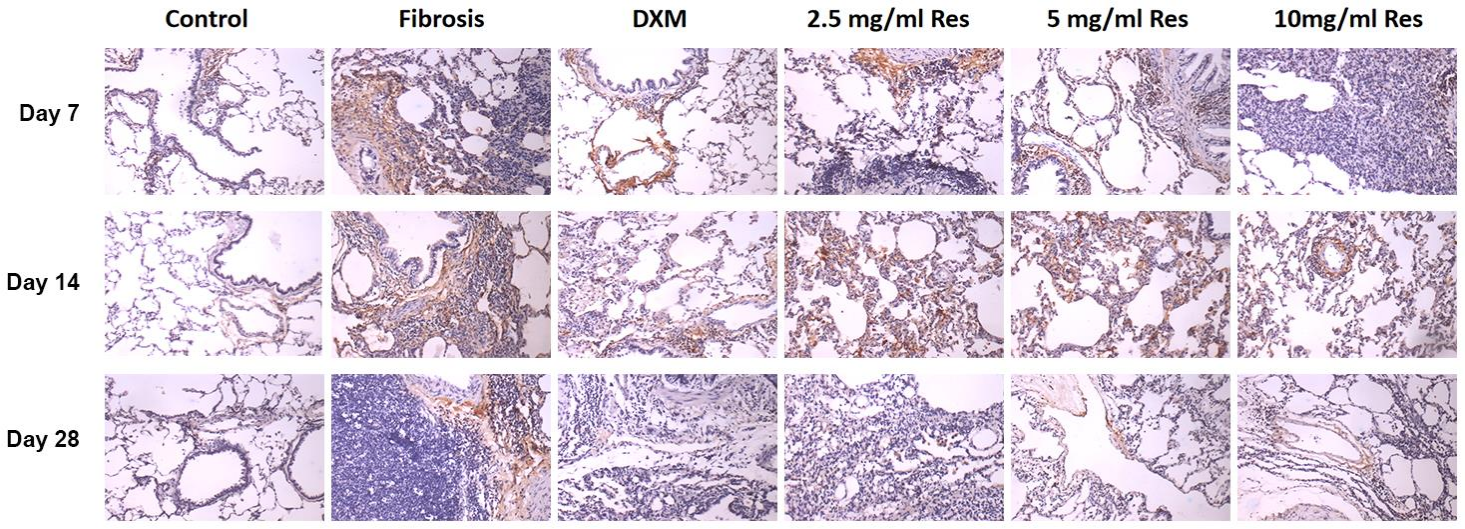
**HIF-1α was overexpressed in the rat fibrosis model.** The expression of HIF-1α was measured by immunohistochemistry on days 7, 14, and 28 following different treatments. Scale bar = 200 μm.

Under normal physiological conditions, HIF-1α is weakly expressed in lung tissues. It was expressed around small blood vessels, and in some inner macrophages and inflammatory cells in the lung interstitium in the model group, mainly located in the cytoplasm. The number of HIF-1α-positive cells in the model, high-Res, middle-Res, low-Res, and DXM groups was higher than that in the control group. Their number was markedly different between the model group and low-Res, middle-Res, and high-Res groups, and the DXM group on day 7 (*p* < 0.05). However, no difference was observed in HIF-1α-positive cells between middle-Res, high-Res, and DXM groups on day 7 (*p* > 0.05). No difference was observed between low-Res, middle-Res, and model groups on day 14 (*p* > 0.05). The expression of HIF-1α in the high-Res and DXM groups was weaker than that in the model, low-Res, and middle-Res groups on day 14 (*p* < 0.05). However, no difference was observed between DXM and high-Res groups on day 14 (*p* > 0.05). Similarly, no major difference was observed between different Res intervention groups on day 28 (*p* > 0.05), and this difference was less than that in the model group (*p* <0.05) and higher than that in the DXM intervention group (*p* < 0.05; Figure 4 and Table 1).

**Table 1:** HIF-1α expression in lung tissue from rats in each group (average optical density value mean ± standard deviation).

**Group**	**7d**	**14d**	**28d**
Control	2.45±0.13	2.40±0.11	2.39±0.21
Model	10.33±0.31*	17.65±1.82*	13.48±1.45*
Low-Res	8.14±0.40*^Δ^	16.62±1.49*	9.09±1.18*^Δ^
Middle-Res	6.38±1.06*^Δ^	17.08±2.25*^Δ^	8.96±1.01*^Δ^
High-Res	6.05±1.20*^Δ^	14.29±1.50*^Δ^	7.91±1.11*^Δ^
DXM	5.64±1.34*^Δ^	13.52±1.23*^Δ^	5.84±1.12*^Δ^

*: Compared to the control group *p*<0.05, Δ: Compared to the model group *p*<0.05.

### NF-κB expression increases in rat fibrosis model

**Figure 5:**
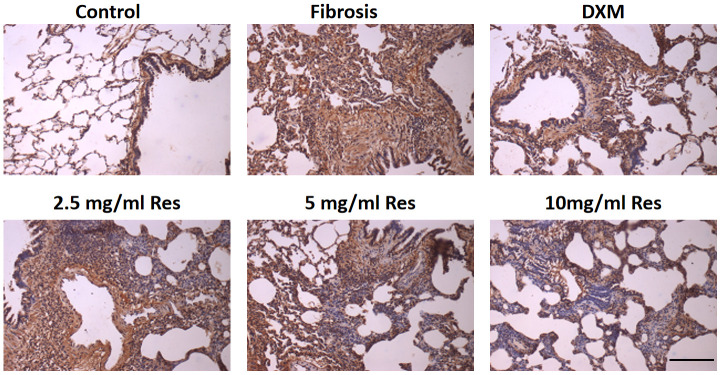
**Increased expression of NF-κB in the rat fibrosis model was detected via IHC.** The expression of NF-κB was measured by immunohistochemistry in the six groups following different treatments. Scale bar = 200 μm.

Under normal physiological conditions, NF-κB is weakly expressed in the lungs. The positive light brownish yellow staining primarily around the bronchi and in certain interstitial macrophages and inflammatory cells was evidence of NF-κB expression. The number of NF-κB-positive cells in the model, high-Res, medium-Res, low-Res, and DXM groups was higher than that in the control group. Reduced expression of NF-κB was observed in middle-Res, high-Res, and DXM groups than in the model and low-Res groups on days 7, 14, and 28 (*p* < 0.05). We did not find any difference in the expression of NF-κB between middle-Res, high-Res, and DXM groups on days 7, 14, and 28 (*p* > 0.05; Figure 5 and Table 2).

**Table 2:** NF-κB expression in lung tissue from rats in each group (average optical density value mean ± standard deviation).

**Group**	**7d**	**14d**	**28d**
Control	2.89±0.19	3.01±0.33	2.90±0.28
Model	28.02±1.36*	42.22±1.29*	54.33±0.58*
Low-Res	27.61±1.28*	42.16±0.99*	53.86±0.76*
Middle-Res	23.67±0.59*^Δ^	28.38±0.64*^Δ^	34.28±1.08*^Δ^
High-Res	23.04±1.46*^Δ^	27.80±1.48*^Δ^	33.92±0.96*^Δ^
DXM	22.14±2.14*^Δ^	27.22±1.89*^Δ^	33.18±1.44*^Δ^

*: Compared to the control group *p*<0.05, Δ: Compared to the model group *p*<0.05.

### Inhibition of HIF-1α partially reverses BLM-induced fibrosis

**Figure 6:**
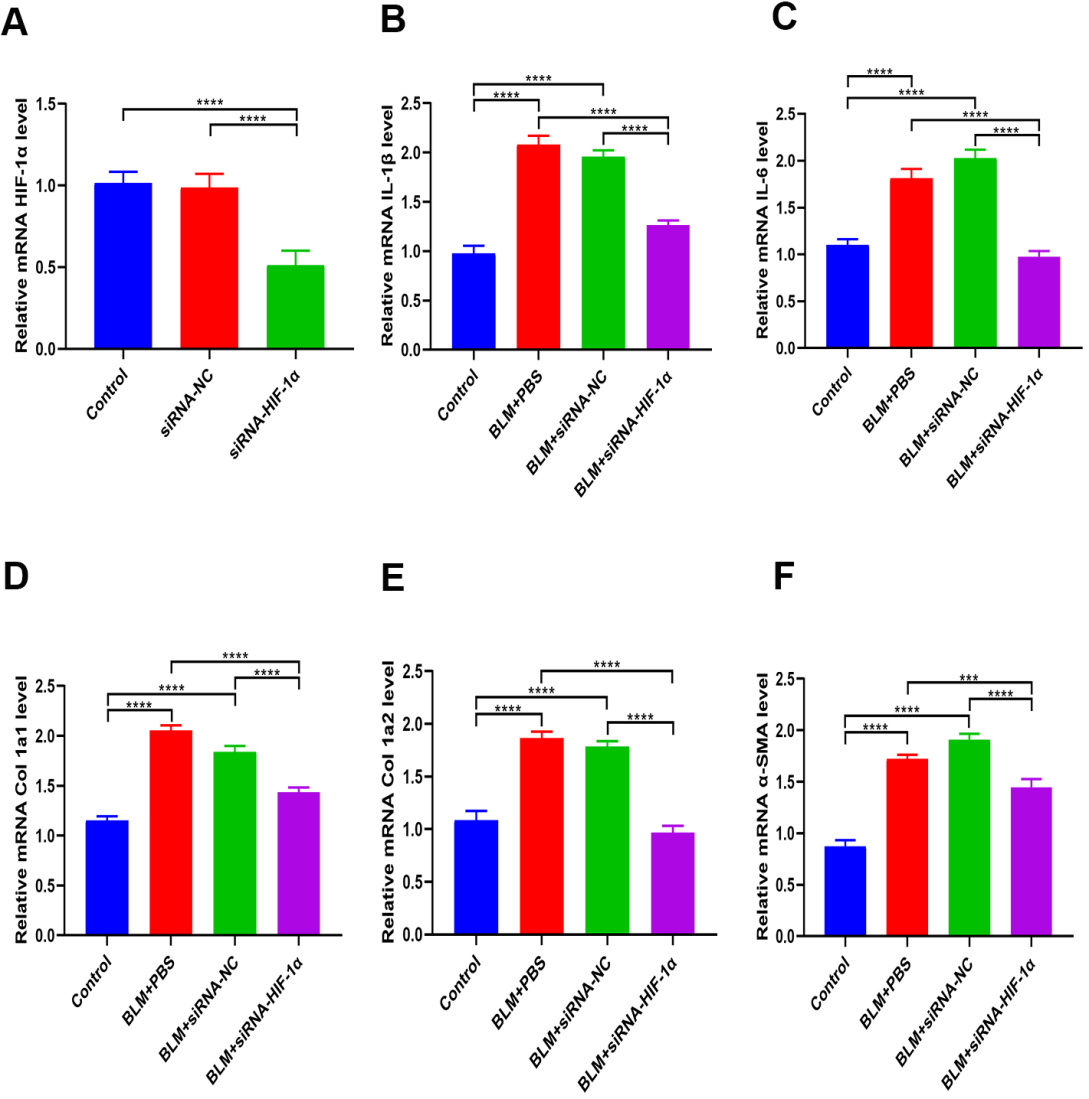
**Inhibition of HIF-1α partially reverses BLM-induced fibrosis.** (**A**) The expression of HIF-1α in BEAS-2B cells with and without siRNA-HIF-1α transfection was measured by qRT-PCR. (**B**, **C**) The expression of inflammatory cytokines in different groups was measured by qRT-PCR. (**D**–**F**) The expression of fibrotic genes in different groups was measured by qRT-PCR.

Expression of HIF-1α in BEAS-2B cells with and without siRNA-HIF-1α transfection was measured with qRT-PCR. The results showed that HIF-1α levels in the cells expressing siRNA-HIF-1α was significantly lower than in the control and siRNA-NC groups (Figure 6A). Moreover, the *in vitro* results suggested that the high levels of fibrotic (Col 1a1, Col 1a2, α-SMA) and inflammatory (IL-1β, IL-6) markers induced by BLM were significantly reduced after transfection of siRNA-HIF-1α (Figure 6B–6F).

### Resveratrol ameliorates bleomycin-induced pulmonary fibrosis via regulation of HIF-1α and NF-κB expression

**Figure 7:**
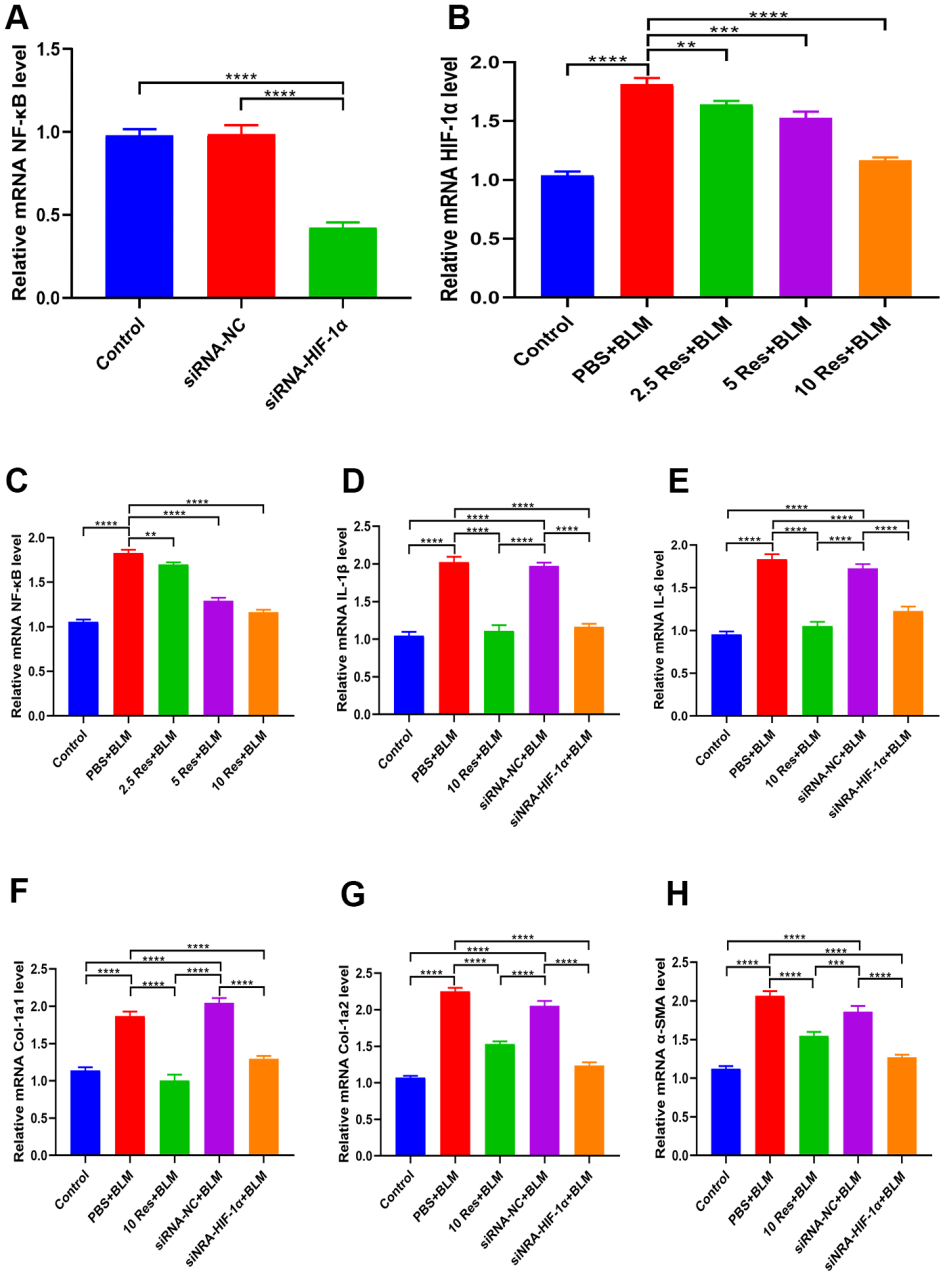
**Resveratrol ameliorates bleomycin-induced pulmonary fibrosis by suppressing HIF-1α and NF-κB expression.** (**A**) The expression of NF-κB in BEAS-2B cells with and without siRNA-HIF-1α transfection was measured by qRT-PCR. (**B**, **C**) The expression of HIF-1α and NF-κB in BEAS-2B cells following different treatments was measured by qRT-PCR. (**D**, **E**) The expression of inflammatory cytokines in different groups was measured by qRT-PCR. (**F**–**H**) The expression of fibrotic genes in different groups was measured by qRT-PCR.

As shown in Figure 7A, qRT-PCR analysis revealed that NF-κB levels were significantly decreased in BEAS-2B cells expressing siRNA-HIF-1α. Likewise, resveratrol suppressed HIF-1α expression in BEAS-2B cells (Figure 7B), and there was a concomitant decrease in NF-κB levels (Figure 7C). Moreover, BLM-induced expression of the inflammatory markers IL-1β and IL-6 was significantly reversed by resveratrol or siRNA-HIF-1α (Figure 7D, 7E). Similarly, BLM-induced expression of the fibrotic markers α-SMA, Col 1a1, and Col 1a2, was significantly reversed by resveratrol or siRNA-HIF-1α (Figure 7F–7H).

## DISCUSSION

HIF-1 is a hypoxia-responsive transcription factor in mammalian cells. HIF-1α, the regulatory and active subunit of HIF-1, regulates hypoxia-induced gene expression, angiogenesis in hypoxic tissue, cell proliferation and survival, iron metabolism, erythropoiesis, and glycolysis [12]. We found elevated expression of HIF-1α in the lung tissues of rats following BLM-induced pulmonary fibrosis, mainly localized to around small blood vessels, inner macrophages, and a few inflammatory cells in the lung interstitium. Moreover, the dynamic change in the HIF-1α expression, which first increased and subsequently decreased, suggested its involvement in hypoxia and inflammatory damage observed in the early stages of BLM-induced pulmonary fibrosis.

NF-κB, a well-studied nuclear transcription factor, is activated by several exogenous or endogenous stimuli. Upon activation, it translocates to the nucleus to induce the transcription of genes involved in inflammation [13]. Moreover, it participates in the pathogenesis of various lung diseases such as bronchial asthma, acute lung injury, acute respiratory distress syndrome (ARDS), systemic inflammatory response syndrome, and occupational lung disease. A gradual increase in the expression of NF-κB in parallel with the development of inflammation and lung fibrosis suggested its involvement in the pathogenesis of pulmonary fibrosis. A concomitant elevation in its mRNA expression in the model group was associated with increased collagen synthesis, which further strengthened the function of NF-κB in causing acute lung injury.

Resveratrol (3,4,5-trihydroxy-trans-stilbene, C_14_H_12_O_3,_ a molecular weight of 228.25 g/mol) is a non-flavonoid polyphenol widely found in 72 seed plants [14]. Because of its varying biological and pharmacological activities, such as anti-oxidation, antibacterial, anti-inflammatory, growth inhibitory, anti-platelet aggregation, pro-apoptotic, anti-tumor, anti-cardiovascular disease, liver protection, and estrogen regulation [8, 15, 16], it is known to exert a therapeutic effect on several tumors, pulmonary fibrosis, and other proliferative disorders [8, 15, 16]. Moreover, resveratrol can inhibit lipid peroxidation, reduce cell proliferation, and preserve the cell membrane integrity against lipid peroxidation-induced damage. In addition, resveratrol can scavenge free radicals and activate antioxidant enzymes, such as superoxide dismutase (SOD), glutathione peroxidase (GPx), and glutathione reductase (GSSG-R), to maintain the dynamic balance of intracellular glutathione, thereby improving pulmonary fibrosis [8]. For example, resveratrol can inhibit fibrosis caused by abnormal remodeling of the extracellular matrix by increasing the SOD activity, reducing the malondialdehyde (MDA) content in lung tissue, and removing excessive oxygen free radicals. It exerts beneficial effects in the later stages of pulmonary fibrosis by regulating the metabolism of collagen in the lung tissues and reducing its deposition in the lung interstitium. Furthermore, by inhibiting the activation of NF-κB in macrophages and lymphocytes, it can reduce the level of nitric oxide as well as those of inflammatory mediators such as tumor necrosis factor-α (TNF-α), interleukin-1β (IL-1β), interleukin-6 (IL-6), transforming growth factor-β (TGF-β) and TNF in pulmonary fibrosis to protect against oxidative damage [11, 17–19]. In that context, we conclude from our results that lung epithelial cells, macrophages, and lymphocytes are all involved in the complex process of pulmonary fibrogenesis.

## CONCLUSIONS

**Figure 8:**
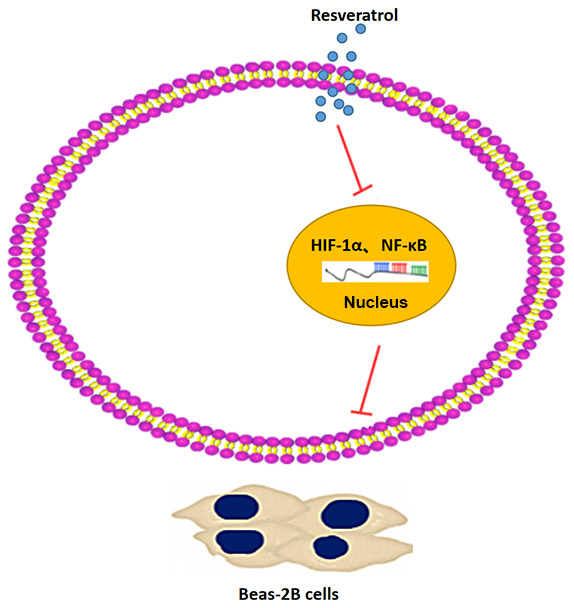
**A schematic representation of the proposed mechanism showing that resveratrol alleviates pulmonary fibrosis by inhibiting the expression of HIF-1α and NF-κB.**

Based on the results of our study, we conclude that resveratrol reduces the inflammatory response and lung damage in pulmonary fibrosis by inhibiting the expression of HIF-1α and NF-κB (Figure 8). Thus, resveratrol could serve as a promising therapeutic drug for pulmonary fibrosis.

## MATERIALS AND METHODS

### Cell culture, cell viability, and transfection

BEAS-2B cells were cultured in Dulbecco's Modified Eagle's medium (DMEM) (catalog D5796, Sigma-Aldrich, MA, USA) containing 10% fetal bovine serum (catalog B9398, Sigma-Aldrich), 100 U/mL penicillin, and 100 μg/mL streptomycin at 37° C and 5% CO_2_. Cell viability was measured using the cell counting kit-8 (CCK-8) assay (catalog ab228554, Abcam, Cambridge, UK) according to the manufacturer’s instructions. BEAS-2B cells were treated with phosphate-buffered saline (PBS), 2.5 mg/mL Res, 5 mg/mL Res, or 10 mg/mL Res for 24 h. Next, CCK-8 solution was added to wells, and cells were incubated for 3 h at 37° C, following which the absorbance was measured at 450 nm. For siRNA transfection, cells were transfected with 50 nM siRNA-NC or siRNA-HIF-1α (GenePharma, Shanghai, China) using Lipofectamine 3000 (Thermo Fisher Scientific, MA, USA) following the manufacturer’s instructions.

### Real-time quantitative reverse transcription-polymerase chain reaction

TRIzol reagent (Thermo Fisher Scientific, Inc.) was used to isolate total RNA from cell and tissue samples. The purified RNA was reverse transcribed into cDNA using the ReverTra Ace qPCR RT Master Mix (Toyobo Life Science) according to the manufacturer’s instructions. The relative miRNA expression was normalized to that of the internal control (β-actin) and was calculated according to the 2^-ΔΔCq^ method. All experiments were conducted in triplicate, and the primer sequences are displayed in Table 3.

**Table 3:** Primers used in the experiments.

**Gene name**	**Primer sequence**
Cyclin D1 - Forward	5’-TTGCCCTCTGTGCCACAGAT-3’
Cyclin D1 - Reverse	5’-TCAGGTTCAGGCCTTGCACT-3’
Cyclin D3 - Forward	5’-CTGGCCATGAACTACCTGGA-3’
Cyclin D3 - Reverse	5’-CCAGCAAATCATGTGCAATC-3’
HIF-1α - Forward	5’-CTACAAGAAACCGCCTATGACGT-3’
HIF-1α - Reverse	5’-CTCAACCCAGACATATCCACCTC-3’
NF-κB - Forward	5’-TGACCCCTGTCCTCTCGCAT-3’
NF-κB - Reverse	5’-GGTCTCGTAGGTCCTTTTGCG-3’
IL-1β - Forward	5’-AGCTACGAATCTCCGACCAC-3’
IL-1β - Reverse	5’-CGTTATCCCATGTGTCGAAGAA-3’
IL-6 - Forward	5’-ACTCACCTCTTCAGAACGAATTG-3’
IL-6 - Reverse	5’-CCATCTTTGGAAGGTTCAGGTTG-3’
Col-1a1 - Forward	5’-GAGGGCCAAGACGAAGACATC-3’
Col-1a1 - Reverse	5’-CAGATCACGTCATCGCACAAC-3’
Col-1a2 - Forward	5’-GTTGCTGCTTGCAGTAACCTT-3’
Col-1a2 - Reverse	5’-AGGGCCAAGTCCAACTCCTT-3’
α-SMA - Forward	5’-TGTATGTGGCTATCCAGGCG-3’
α-SMA - Reverse	5’-AGAGTCCAGCACGATGCCAG-3’
β-actin - Forward	5’-GTGGGGCGCCCCAGGCACCA-3’
β-actin - Reverse	5’-CTCCTTAATGTCACGCACGATTTC-3’

### Animals

Ninety Sprague-Dawley rats were used in this study. They were randomly divided into control, model, low-Res (25 mg/kg), middle-Res (50 mg/kg), high-Res (100 mg/kg), and DXM (3 mg/kg) groups.

### Ethical statement

Protocols of the present study were approved by the Ethics Committee of Shanghai University of Medicine and Health Sciences Affiliated Zhoupu Hospital.

### Histological scoring

The lung tissues were embedded in paraffin and cut into 5 μm-thick sections. Lung sections were stained with H&E and Masson’s trichrome stain for evaluation of histopathological changes. Pulmonary fibrosis was assessed using Masson’s trichrome staining, and lung fibrosis was scored using Image-Pro Plus 6.0 software (Media Cybernetics, Silver Spring, MD, USA). The histological features of lung sections were graded blindly by three experienced pathologists.

### Immunohistochemistry

Paraformaldehyde-fixed and paraffin-embedded lung tissue sections (5 μm thick) were incubated overnight at 4° C with rabbit antibodies against HIF-1α (1:1000, catalog ab51608, Abcam) and NF-κB (1:1000, catalog ab145954, Abcam). Next, the samples were incubated with goat anti-rabbit IgG (Abcam) for 1 h at room temperature.

### Statistical analysis

The data are presented as mean ± standard deviation (SD). Comparison between two groups was performed using Student’s *t*-test, and that between more than two groups was performed using one-way analysis of variance (ANOVA). Analyses were conducted with GraphPad Prism 8.0. A *p* < 0.05 was considered significant.
